# “Imagine, 7 Years Without a Future”: A Qualitative Study of Rejected Asylum Seekers' Life Conditions in Norway

**DOI:** 10.3389/fsoc.2022.813994

**Published:** 2022-07-19

**Authors:** Mette Sagbakken, Ida Marie Bregaard, Sverre Varvin

**Affiliations:** Faculty of Health Sciences, Department of Nursing and Health Promotion, Oslo Metropolitan University, Oslo, Norway

**Keywords:** migration, rejected asylum seekers, mental health, structural violence, wellbeing

## Abstract

Asylum seekers are in an extraordinary situation as their future life depend on decisions made by authorities in a bewildering, bureaucratic system, with excessive waiting and unpredictable timeframes. Those that are not granted asylum, and not able to return to their country of origin, can neither spatially nor temporally visualize if, when or how a potential change is going to occur. This paper is part of a larger study based on narrative interviews with asylum seekers and refugees in asylum centers in Norway, exploring their experiences before, during, and after flight. As we found that the life circumstances for those being refused asylum, were highly different from other participants in the project, we chose to address this particular group in a separate paper. The participants in this part of the study consisted of 21 individuals (of a total of 78 participants) in the age range 18–44, of whom eight were female and 13 males. Trough qualitative interviews and participant observation the aim of this study was to explore and describe the life condition and mental health situation of rejected asylum seekers in Norway. We found that the gradual loss of rights, opportunities and finances are experienced as a form of violence that leads to extreme mental and social suffering. This policy clearly conflicts with Human Rights incorporated in the Norwegian constitution, and we argue that it legitimizes treating asylum seekers as a group of undesirable and underserving political bodies, with serious consequences for their mental health and wellbeing.

## Introduction

Of the 89,3 million people being forcibly displaced at the end of 2021, around 36,1 million have crossed borders and thus are defined as refugees (United Nation's Refugee Agency, 2022). After the invasion of Ukraine in 2022, the number has increased by around 7,7 million (United Nation's Refugee Agency, 2022). A rigorous stance on accepting asylum seekers in Western nations was outlined in the EU-Turkey Statement from 2016 (European Commission, 2016; Gammeltoft-Hansen, 2016). The EU-Turkey Statement was clearly meant to relieve congestion on Europe's borders and deter future asylum seekers and economic migrants from embarking on the risky voyage. It was also intended to send a message—both publicly and internally—that EU Member States could stand unified on issues that cut to the heart of the union. The agreement was one of numerous attempts at the time to limit migration to Europe; restrictions in the Western Balkans migratory route were also important. This approach has had direct repercussions, such as enhanced border surveillance and border closures, as well as indirect consequences, such as higher risks of injuries and health damages, sexual assaults, and death during flight (Eleftherakos et al., 2018; Kingsley, 2018; Kien et al., 2019; Maldari et al., 2019).

The unprecedented number of asylum seekers in 2015–16 also prompted Norway to impose new restrictions. In 2015, the number of asylum seekers who arrived in Norway nearly tripled compared to the previous year, and a list of restrictive measures [Prop. 90 L (2015–2016)] to complicate settlement and family reunification was proposed. Norway's stated political purpose was to avoid seeming overly tolerant in comparison to other European destination countries (Eggebo and Brekke, 2019).

This study is about rejected asylum seekers' life conditions in Norway. In general, it is well documented that asylum seekers and refugees have greater rates of mental health problems than host communities (Priebe et al., 2010; Bogic et al., 2015; Silove et al., 2017; Opaas et al., 2020). The upheavals associated with the refugee experience also have serious impacts on developmental paths and especially on identity development (Varvin et al., 2021). Psychological development is a lifelong process, and being forced to flee, often accompanied by severe traumatization, implies ruptures in development at all ages that can be difficult to heal (Arja, 2014; Ball and Moselle, 2016).

Most participants in our study had to rely on human smugglers to get them out of destitute and often war-torn parts of their home country. Their flight experiences have been full of suffering, often life-threatening situations, a lack of coverage of basic needs, dehumanizing conditions, and experiences of loss. As a rule, arrival in a country where they can seek asylum represents a situation filled with expectations and hope for safety, care, understanding and for possibilities for their future life. Entering a system governed by bureaucratic rules which at face value are not adapted to their psychological situation and which can objectively accept or reject their needs is often experienced as a repetition of their earlier cruel and dehumanizing experiences (Varvin, 2017, p. 173), and increases the risk of mental illness (Hocking et al., 2015). The situation of a long wait or rejection is often described as the worst part of their journey, even worse than prison and torture, especially when their hopes gradually or suddenly are dashed by prolonged waiting or rejection of their asylum application (Sagbakken et al., 2020).

More than anything, waiting also characterizes the refugee experience before and during flight. It is a period of hope, but a hope that cannot be anchored in a defined future experience; a period expressed by several of the participants in this study as an endless wait. The word “wait” has thus lost its meaning because to wait implies waiting for something to happen, someone to come, for a time for departure, for a meal and, for many, for a decision on their asylum application (Bjertrup et al., 2018). The term “liminal phase” is often used for the stage in a “rite of passage” where the subject is on the threshold of a new and partly unknown time period (Turner, 1964, p. 4–20). It is often described as the transition period between two stages where the next is more or less known, like a “passage of transition” between youth and adulthood (Turner, 1964). Similarly, the concept of *limbo*, (lat. *limbus*) means “border” or “in between”. It refers to an intermediate state or condition (i.e., liminal space) or a situation being so uncertain that it is perceived as beyond one's control; and in which there is no improvement or progress in sight (Hartonen et al., 2021). Both limbo and liminality have been used widely in the contemporary research literature, as refuges and asylum seekers can be said to exist “outside the natural order of things” or being in a state of exception; including exclusion from society, existence outside the law, and being deprived of functions and basic human rights. Even though there are subtle differences between conditions in transit, in camps and in reception centers, legal liminality is a shared attribute of them all (Hartonen et al., 2021).

In this article, we will present the experiences of rejected asylum seekers who live or have lived in reception centers in Norway. The study addresses these participants overall life situation, and thus health in a broad term as outlined by WHO; “a state of complete physical, mental and social wellbeing and not merely the absence of disease or infirmity.” (World Health Organization, n.d.).

### Health Problems of Finally Rejected Seekers and Other Undocumented Migrants

The individual migrant's health is the result of, inter alia, previous experiences, the migratory process, social determinants of health as well as the migrant's current social situation (De Vito et al., 2015). Several international studies show that health problems among undocumented migrants/rejected asylum seekers correspond with the health problems seen in ordinary general practices. Most of the health problems are mainly related to digestive problems, mental health issues, musculoskeletal disorders and obstetric care, and can be treated in primary health care (Ehmsen et al., 2014; The City Church Mission, 2021). Some infectious diseases, e.g., tuberculosis and hepatitis, are more prevalent among undocumented migrants than among the general population (De Vito et al., 2015). Relatively few studies differentiate between undocumented migrants in general and finally rejected asylum seekers. Those that do find that the latter group has a high prevalence of mental disorders and suicidal ideation (Mueller et al., 2010; Schoretsanitis et al., 2018) and most have experienced several traumatic life events (Opaas and Varvin, 2015; Shannon et al., 2015). Finally rejected asylum seekers also experience high stress levels and deteriorating health aggravated by a negative asylum decision (Mueller et al., 2010; Morgan et al., 2017).

The fact that finally rejected asylum applicants are not able to work or study due to legal restrictions can also be seen as a source of health deterioration. Further, they rarely manage to participate in events in their local communities or even at the reception center due to financial constraints (Halogen, 2018). When children are involved, they are offered kindergarten places from the age of four, while some municipalities or reception centers offer day care for children aged between two and four (Halogen, 2018). All children must attend primary and secondary education if they are expected to stay more than 3 months. However, as finally rejected asylum seekers tend to live in poverty, their children are prevented from participating in most extracurricular activities, and most of them stay at home with their parents most of the time (Weiss, 2013; Seeberg, 2017). Many have severely ill parents with no or little possibility of receiving treatment (Royal Decree on services for people without permanent residence, 2011). Thus, finally rejected asylum-seeking children are in a very vulnerable situation, as they are excluded from extracurricular activities while at the same time they might be the only ones in their family who learn to speak Norwegian and who are part of society to a certain extent (Weiss, 2013). A report commissioned by the Norwegian Ministry of Justice and the Police found that parents with failed asylum applications tend to prioritize the needs of their children over spending money on, for example, medicines for themselves (Lidén et al., 2011). Even so, several of these children lack basic necessities such as winter clothing (Halogen, 2018).

### Rights to Health Care for Rejected Asylum Seekers

Asylum seekers are a part of the national insurance scheme and have the same rights and access to health care as the general population. Most municipalities have designated health services for asylum seekers. Rejected asylum seekers have very limited rights, however. The United Nations Declaration of Human Rights envisages the highest attainable standard of health as a fundamental right of every human being (United Nation's General Assembly, 1948). In spite of the European Convention on Human Rights being incorporated into Norwegian legislation, the regulations for health and social services for persons without legal residence (Royal Decree on services for people without permanent residence, 2011) states that “People without legal residence in Norway are only entitled to medical care that cannot wait” and that this is understood as:

*health care that is absolutely necessary and cannot wait without danger of imminent death, permanently impairment, serious injury or severe pain. If the person is mentally unstable and constitutes an obvious and serious risk to their own or another's life or health, the person is entitled to mental health care regardless of legal status*. (Royal Decree on services for people without permanent residence, 2011)

Children have almost full rights to health care, but do not have the right to be registered with a general practitioner (GP) (Royal Decree on services for people without permanent residence, 2011). Despite having the right to some health services, rejected asylum seekers are often asked to pay for the services rendered. This includes, inter alia, costs for prenatal and postnatal care, which is free for members of the National Insurance Scheme. There are examples of women having to pay approximately EUR 25,000 for pregnancy-related care (Brandvold, 2021). Both the reduced right to health services for rejected asylum seekers and patients having to pay for the services have been criticized by the (United Nation's Committee on Economic Social Cultural Rights, 2020). Due to the large demand for health care in this group, a health center for undocumented migrants was established in 2009 in Oslo, the capital of Norway. This center provides health care free of charge to all groups of undocumented migrants, and most of the patients are rejected asylum seekers (The City Church Mission, 2021).

### The Norwegian Asylum Process

According to the Universal Declaration of Human Rights, Article 14, all people have the right to seek asylum in the country of their choosing (United Nation's General Assembly, 1948; Norwegian Directorate of Immigration, n.d.-b). The right entitles the applicant to a consideration of the application after the current criteria but does not grant protection (asylum). The number of asylum seekers to Norway varies, from 31,145 applicants during the increased refugee influx in 2015 to 1,386 applicants in 2020 (Norwegian Directorate of Immigration, 2016). The number of applicants mainly depends on factors outside of Norway, such as the EU–Turkey Statement (European Commission, 2016). The current average waiting time for receiving an answer to an application in Norway is 1,600 days, or ~4 years and 4 months. This partly depends on the country of origin and the reason for applying for asylum (Weiss et al., 2017; Haugland, 2021). During this period, asylum seekers have the right to stay in the country and are offered free, voluntary accommodation at an asylum reception center (Norwegian Directorate of Immigration, n.d.-a). However voluntary, this offer might be their only option, since asylum seekers lack both financial and social resources (Weiss et al., 2017). A study of the housing and living conditions of the asylum reception centers showed that half of the centers had several factors that negatively affected the inhabitants' quality of life and their mental health (Grønseth et al., 2016; Strumse et al., 2016), such as their remote location and crowded conditions. In many reception centers, the inhabitants share bathrooms and kitchens. Some asylum seekers, including families with children, have only one room, with no space for a dining table, and they have to eat on their beds or on the floor (Lillevik and Tyldum, 2021).

In December 2020, there were 1,757 asylum seekers living in Norwegian asylum reception centers. Approximately 40% of the inhabitants came from Ethiopia, Eritrea, and Iran and more than half of these groups had received a final rejection (Norwegian Directorate of Immigration, 2021a). Of the 1,757 applicants, 236 had applied for asylum but had not yet received an answer, 266 had a pending appeal with the immigration authorities, and 729 had received a final rejection. If an asylum application is rejected, there is an opportunity to appeal two times. After a final rejection, asylum seekers are given a short deadline for leaving the country, though they may stay in the asylum reception center for the duration of their stay in Norway.

A finally rejected asylum seeker can apply for assisted voluntary return and reintegration via the International Organization for Migration (IOM) and receive assistance in their home country (International Organization for Migration, n.d.). Not all finally rejected asylum seekers accept voluntary return out of fear of persecution if they return to their home country. The immigration authorities can forcibly return finally rejected asylum seekers to their country of origin if the country accepts them. The police may detain the rejected asylum seekers at the National Police Immigration Detention Centre, and while most stays last for <72 h, the Police can detain a person for up to 18 months (The Norwegian Police, 2022). The Detention Centre has received massive critique over the last years due to several examples of self-harm and suicide attempts (Norwegian Broadcasting Corporation, 2022).

However, many come from countries representing a paradoxical agreement among the government parties. For a variety of reasons, such as being stateless or not having an extradition treaty with the country in question, the person cannot be subject to forced return (known as “unreturnable”). In other words, they are “allowed” to stay but lose their right to work or education, and the right to social benefits.

There have been various barriers to both voluntary and forced return for some groups, such as Eritreans and Ethiopians with final rejections, resulting in hundreds of these nationals living in reception facilities for several years after getting a final denial (Norwegian Directorate of Immigration, n.d.-c). The Norwegian Immigration Police has a list of 2,550 “undocumented migrants”, of whom 2,250 are finally rejected asylum seekers who are to be apprehended and returned to their country of origin (Norwegian Police Service, n.d.). Finally rejected asylum seekers are not allowed to study or work and receive a monthly benefit from the Norwegian Directorate of Immigration (UDI) of NOK 1,992 (EUR 196) (Norwegian Directorate of Immigration, 2021b) for the duration of their stay at the asylum reception center. This is 18% of the Norwegian reference budget for consumer expenditure, and the amount is meant to cover the asylum seeker's general expenses, including food, sanitary products, and medical expenses. However, this monthly benefit has decreased over the past 10 years, even though the general price level has risen. The consequences are, among others, food insecurity (Henjum et al., 2019) and lack of access to health services because they cannot afford transport or doctor's fees (Halogen, 2018; Obtinario and Thorkildsen, 2020).

Unaccompanied minor asylum seekers with a final rejection cannot be returned before they turn 18 years of age. Some move from a reception center for minors to a regular reception center when they turn 18, but they experience challenges that differ from those faced by adults due to their age specific developmental situation. Many lose hope and have problems finding a meaning in life while living in limbo. For a young person, the concept of waiting can be more difficult, as 1 year or more is often perceived as much longer compared to people in their thirties (Øverland et al., 2020).

## Theoretical Frame

As a response to the empirical material, this paper focuses on structural violence in the understanding of Galtung's (1969) early work. We find this frame a useful point of departure as the empirical material describes mental and social suffering due to a form of violence, even though the violence is not physical and may not even be visible. However, as emphasized by Galtung (1969, p. 168), “Violence is present when human beings are being influenced so that their actual somatic and mental realizations are below their potential realizations”. Thus, this type of violence can be seen as the cause of the discrepancy between the potential and the actual possibilities to meet physical, social, and mental needs. This goes beyond the distinction between violence that works on the body and violence that works on the mind, since this type of violence does not necessarily imply being hurt somatically but creates conditions that place constraints on the physical body. It cannot move, travel, exercise or play freely due to economic or juridical restrictions. Similarly, even though the person may be treated in a way that may comply with government or local directives, the unpredictability and insecurity related to where the physical body is to be placed may imply psychological torment or mental violence, as articulated by Galtung (1969). Galtung also discusses whether there can be violence when there are no visible subjects that directly execute the violence. In the case of the material in our study, the participants rarely experience violence that can be traced back to single actors, but the violence is indirect and structural, not least *because* those who decide are invisible, difficult to reach and part of a large, distanced, and bewildering system. Thus, the violence is built into the system and manifests in feelings of insecurity, powerlessness, and a sense of being dehumanized. This correlates with Galtung's description of unequal power to decide over the distribution of and access to resources as structural violence.

Asylum seekers have limited access to means to realize their actual potentials and rejected asylum seekers experience a systematic decrease in such opportunities throughout their process of applications and appeals. The situation is often aggravated when people are low on income, education, health and power, which is regularly the case as these variables tend to be heavily correlated in all social systems (Galtung, 1969). As the situation deteriorates and individuals are deprived of opportunities, including hope of future opportunities, their mental health may be affected.

An important question is whether the violence is intended or not (Galtung, 1969); a question where the answer is not straightforward when interpreting the narratives in this material. However, as will be further elaborated in this paper, the perception that there is a punitive element in the gradual de-escalation of rights and opportunities among this group of asylum seekers is pronounced.

Citizenship, described as being a “complete member of society” by Marshall (1950), is a related framework to be employed in the interpretation of the data. Citizenship is described as the ability to act in a constructive manner toward other members of the community, such as being a good neighbor, and comprises a set of rights ranging from economic stability to the right to share and live the life of a civilized human in accordance with societal standards. According to Marshall (1950), it also covers the right to education, health care, and all other areas of social welfare. Being a true citizen also includes having equal civil and political rights, enabling everyone to influence his or her own situation—all in line with the ideas behind the Universal Declaration of Human Rights.

## Methodology

This qualitative study from Norway is part of a larger mixed-method study with participants recruited in both Norway and Serbia. The aim of the larger study was to identify resilience-promoting and resilience-inhibiting factors on both individual and contextual levels for asylum seekers during their stay at asylum reception centers in Norway and during their journey through the Balkans.

The qualitative part of the overall study was explorative, and a short, open, semi-structured interview guide was developed. The guide consisted of three main questions, created to gather the participants' narratives describing difficult and helpful pre-flight, flight and post-flight experiences, including their perceived quality of life from the time they decided to leave their home until the interviews were conducted. During the analysis of the data in both the Norwegian and Serbian context, several main subthemes emerged, and one of the prominent topics that emerged from the Norwegian qualitative data was the difficult living situation of rejected asylum seekers (most of the Serbian respondents did not apply for asylum in Serbia; the country mostly serving as a transit country). Through the analysis of the Norwegian quantitative data, we found that the mental health of the participants that were refused (rejected application) was significantly worse than that of the others (Grøtvedt et al., 2022).

### Recruitment and Sampling Strategy in the Norwegian Sample

In terms of the overall study, a purposeful sampling strategy, aimed at maximum variation, was applied to obtain as many different perspectives as possible, including variations in age, gender, family situation, education level, and ethnic affiliation. This ensured inclusion of the subgroups of families with children, single mothers with small children, single women, asylum seekers arriving as unaccompanied minors but tested to be adults (above 18 years) and refused asylum seekers/refused asylum seekers in the process of appealing. Some of the participants were aged below 18 (minors) when they arrived in Norway but had turned 18 while waiting for their application to be processed. Asylum seekers from Syria, Iraq and Afghanistan were of particular interest due to their common flight experiences, but all asylum seekers were invited to participate. The inclusion criteria were asylum seekers living in asylum reception centers above 18 years of age. The exclusion criteria were asylum seekers below 18 years of age or too ill to be interviewed.

In terms of the Norwegian part of the overall study, 78 participants were recruited at five reception centers for families and single adults in different counties. The sample included rural and urban receptions centers, including regions far from the capital, as we believed that there would be differences in the reception center organization and the social context around the participants (Patton, 1990). The participants all perceived themselves as refugees and all had applied for asylum (47% application pending, 24% granted asylum, and 29% refused asylum). Length of stay in Norway varied from 3 months to 10 years. Since we found that the situation of this group was quite different and included more serious psychological consequences, we chose to address this group in a separate paper.

### Characteristics of Participants in the Present Norwegian Sub-study

The participants in this Norwegian sub-study consisted of 21 individuals in the age range 18–44 (mean age 29), of whom eight were female and 13 males. They had all had their asylum application rejected; however, some were in the process of appealing (you can appeal twice). Sixteen of the participants were single, and five were married/had a family. Twelve of the participants had children, while nine had not. They came from countries located in the Middle East, East-Africa, and other sub-Saharan African countries. Four of the participants had education at university level, while 10 had completed high school, and seven had completed primary school. In the quantitative part of the study (Grøtvedt et al., 2022), the Hopkins Symptoms Checklist (HSCL-25) (Mollica et al., 2004), which measures symptoms of anxiety and depression, 17 out of 21 of the participants exhibited clinically significant anxiety, and 18 out of 21 qualified for major depression. According to the Harvard Trauma Questionnaire (HTQ) (Mollica et al., 1992, p. 111–116), which measures a variety of trauma events as well as emotional symptoms considered to be uniquely associated with trauma, eight qualified for PTSD (4 missing).

### Data Production

The data production lasted for 18 months, from 2016 to 2017. The interviews were conducted by three researchers with different backgrounds—one from psychiatry, one from nursing and one from nursing/anthropology—and four master students within the field of nursing, under supervision. Interpreters were used, when necessary, by phone or in person (English and Norwegian were used without interpreters when possible and when requested by the participant). Interviews took place in a quiet room at the asylum reception centers and lasted between 1 and 3 h. All interviews were tape-recorded with the participants' consent, and transcribed verbatim. Notes were taken during the interviews and gave information about the context, the atmosphere during the interview, body language, and researchers' reflections following the interview.

The research included participant observation and field notes in the selected reception centers. This contextual knowledge facilitated a better understanding of what had been expressed during interviews (Fangen, 2010), and enabled us to go beyond selective perceptions and discover issues that were overlooked during interviews. Furthermore, spending time with the residents by participating in activities contributed to build rapport and facilitated conversation as well as formal interviews.

### Ethics

Ethical approval for the study was obtained from the Regional Committee for Medical Research Ethics (REK) in Norway (REK 2016/65), and informed written consent was obtained from all participants. The participants were told that all information would be treated without disclosing names or any other direct identifiable information, and that we would present the data in a way that ensured confidentiality. It was underlined that the research had no link to the asylum application process, and that they could withdraw at any stage. De-identification and confidentiality were ensured by using fictitious names or numbers, and other potential identifiers were also altered.

### Analysis

The data analysis was inspired by the principles of Giorgi's (1985) phenomenological analysis as modified by Malterud (2017). This analytical approach attempts to understand the meaning of events and interactions within the framework of how individuals make sense of their world. We developed an approach that relied on six interconnected stages: (a) familiarization, (b) indexing, (c) identification of a thematic framework, (d) interpretation: development of preliminary categories, (e) confrontation with relevant theory, and (f) reinterpretation of themes, contextualization, and development of the final conceptual (or categories) framework.

As the main researchers (authors of this article) conducted many of the interviews (in addition to master students), familiarization began during the interview process and was followed by in-depth reading of the interviews immediately after they were conducted. We all (including the master students) wrote down our first impressions of and reflections on the interview in the form of reflective notes that were available to all the authors. To ensure consistency within the employed analytical procedures, identification of meaningful units and themes (indexing) was done through reading and re-reading the interviews and notes in a collaborative process between the first and last author. Thus, this study is also inspired by hermeneutics, as we have obtained a constant and dynamic dialog with the material by moving between perceiving and analyzing the material at a detailed level and by the authors trying to understand how the individual pieces constituted broader themes. Through an interpretive process, a preliminary network of categories was developed, clustered around themes based on the focus for this study. In this process new (sub)themes emerged, including themes related to the specific situation of asylum seekers with rejected applications. Finally, the themes were confronted with relevant theory, which included reinterpretation of themes, contextualization, and the analysis moving to a more abstracted level of interpretation. Regarding the situation for people with rejected asylum applications, the following sub-categories were developed: waiting for nothing, loss of identity and social citizenship, access to health care, the double burden of becoming an “adult” and being rejected, losing recognition as a human being. These categories will be elaborated below.

## Findings

### Waiting for Nothing

This study shows how difficult it is to live in an unresolved situation such as seeking asylum. According to the overall data material, the time and the unpredictability involved had other and often more serious psychosocial consequences for those who are rejected asylum.

One of the reasons why this seems evident is that this group of people cannot relate hope of change to any future point in time, since they have no possibility to visualize whether, when or how a potential change is going to occur in the future. Those who have had their application denied but have opted to appeal retain some semblance of optimism, but the progressive erosion of rights and prospects within this group produces a situation with an increasingly bleak future. Regardless of whether the subject had experienced their first or final rejection, being in such a circumstance was reported as exceedingly difficult and was related with emotions of emptiness, powerlessness, and hopelessness.

A woman in her mid-thirties, who came from a region of the world where (according to the Ministry of Foreign Affairs), returning people with refused asylum petitions is extremely difficult, had waited 5 years for her final rejection (including the waiting process for two appeals). After 7 years in Norway, she described her everyday life as without content, and characterized by an existential unrest:

*I take my sleeping medication and go to bed early. And the next day I get up, and after breakfast I take my medication [psychotropic drugs]. Without medication everything is dark. I get worried, I can't manage without medication. Sometimes I think about taking my own life. Imagine, seven years without a future. […] So, the whole day I have nothing to do. I try to comfort myself, and entertain myself, but I can't [pause] So, then it becomes evening again, and the same routine again..*.

When asked about her thoughts about the future, she said:

*Nothing. I think nothing. I don't know what to think. [Pause] I only have one hope… that one day they [the authorities] will realize my situation and give me asylum*.

Several of the participants described the undefined waiting as a situation somehow worse than the situation they were in before they left, even if that situation was characterized by war, persecution, and death. An 18-year-old man from a country at war told how he had adapted to relate to different types of torment by a certain perception of time:

*There are differences between here and my home country. In my home country there is war. […] And there it is like, if something suddenly happens, like if someone dies, then the family are supposed to mourn for three days. And then, yes [pause] then life starts again. But here it is … I don't know…I don't know… I am in a situation with a lot of stress, here it is [pause] you get tormented in a different way. It is worse here than at home..*.

The participant continued to explain how a lack of a time frame and not understanding who the premise provider was had rendered him unable to cope, creating “insanity”:

*I have received my first rejection, but I wait for another answer [second application], and it has been 10 months. For 10 months they have not given me an answer. But why? […] You get totally insane, you get totally insane. Who is UDI? [Norwegian Directorate of Immigration]. I ask everyone, and they tell me ‘No, we can't answer you'. But who can I go to? Who is this UDI? Who is that body, who is that person? You get totally insane, you become totally desperate. […] If a person waits and people says, ‘maybe tomorrow', ‘maybe the day after tomorrow', you are just being left on hold*.

The young man expressed how he saw the body of UDI as an invisible, unreachable, and insurmountable authority that created feelings of alienation and powerlessness among those destined to wait for an undefined period of time.

Similarly, another 18-year-old boy described the psychological pressure of waiting for an answer to the asylum application, and the subsequent feeling of receiving the rejection:

*I came here to get a good life, but everything became worse really, due to the psychological pressure they put you through, it makes you sick*.

*INT: Can you exemplify?*

*OBJ: The waiting. Nothing is worse than the waiting […] [not knowing] How long do I have to wait? […] And then after all this waiting, you get a rejection. It is like climbing a high mountain, and then when you reach the top, someone suddenly throws you down from it*.

The participant described his experience as a feeling of climbing a high and demanding mountain, investing time as well as physical and mental strength. However, when he finally reached the top, he was “rewarded” by being thrown down from it in a brutal manner.

Another participant, a father in his late thirties and his 12-year-old son, waiting for the result of their appeal, described how their daily life felt like being in a prison:

*INT: Can you tell me how your life is now, how a day looks like here at the reception center? Eh, to be honest, during the two years I have been here, it has been like a prison for me. We do get some help, we get food, we get a place to sleep. But it is only eating, going to the toilet, and maybe go out wandering around. [pause]. I have had thoughts about taking my own life. Thinking that it would have been better to be killed by [Islamist movement] than being here*.

When asked about the daily life of his son and the possibilities for activities, he replied:

*No, there is nothing, no activities. We have been at this reception center for one and a half months, and we cannot go anywhere, or do anything. […] There is a TV in some of the rooms at the reception center. […] I asked those working here at the center if we could get a TV […] to watch some cartoon films, since I have a child and he has no other activities, and he gets sad due to that […] We did not get a TV. We only eat and sleep in this room, there is nothing more*.

He described not only an absence of meaningful activities, but also a lack of social arenas that could secure some sort of psychosocial development for his son.

### Loss of Identity and Social Citizenship

A related theme that frequently came up was how the gradual economic and social restrictions made life increasingly difficult to in any way participate in the society as a citizen. One aspect of this was that all the study participants (including those who were still waiting for a decision on their first appeal) reported how the money received was insufficient to pay for patient charges, patient transport to necessary services, medication, healthy food (e.g., fruit and vegetables), hygiene articles, and warm clothes. The situation becomes increasingly worse after the asylum seekers receive their second rejection and final rejection. A man is his early twenties explained his perception of this situation:

*There are two big problems you [the interviewer] must know about. The government don't give people work permits and they know they can't send people back because they know there are problems there. So, what are they waiting for? […] Can you imagine, the first year I had money to pay for food and… […] If you get your first negative [application], you still have a chance to get a little money, like 2,500 kroner a month [EUR 247]. [After the] second negative, it's 900 kroner [EUR 88] every second week. […] Maybe they [UDI] want people to die. They want you to suffer, and to be tired of yourself and say ‘I'm done, drop me home*.

The participant explained how financial support was reduced in line with the likeliness of obtaining a residence permit, the second denial leaving people in a situation very hard to manage everyday life. The same participant elaborated how the loss of his identity card reinforced his difficult situation:

*Now I can't even get medicine when am sick. I have no ID. Even if I show you my ID, it expired in 2012. They can't renew it. So, I go like a dog in the street. When police catch me, I say I live here. When I tell them my name, they look in the computer and see my address, but I have nothing. If you have nothing, even if people send you money, you can't receive it. You can't get anything without ID in this country. NINE YEARS [raising voice] here, and seven years in this situation*.

Individuals lose not only access to long-term economic support, but also the ability to maintain their identity, as demonstrated by this participant. This relates to their ability to document who they are as well as their ability to exercise the rights that make up the society in which they continue to exist. One such example is a young man asking the reception center for a SIM card for his mobile phone, to be able to contact his family back home. He was told “*You are not registered here [Norway] and you have to wait and see what will happen to you”*.

A woman in her mid-thirties, living with her 2-year-old son born in Norway, explained how lack of access to different social arenas was affecting their lives:

*It makes me sad; my son is almost two years old now and he should have been in the kindergarten, but he isn't. We know another family with a child his age […], but they got a residence permit and are settled in a municipality. So, their daughter […] has already been in the kindergarten for a year. So, when I look at my son, I become so sad, because he wants to play, he wants to participate in activities*.

*INT: So, because you do not have a residence permit, you can't access a place in a kindergarten?*

*Actually, there are some people in [other municipality] that are offered a place, but in this municipality, they say my child will not be offered kindergarten before he is four years old. Can you imagine, four whole years, what is he going to do until then?*

She drew attention to not being able to participate in society through work or even Norwegian classes:

*For a human being to feel like a human being, a person must work, manage their own life. It is only animals that are passive recipients, who just exist. Even animals make an effort to access food and while we are just living here and get a minimum to survive, and that is not a life, it is not a life […] I have lived here for seven years now, I should not have been talking through an interpreter, but you know, you don't get access to any language course. […] After the denial came, I had to interrupt, and it [not talking Norwegian] is embarrassing*.

Not being able to participate in the society through playing, studying, or working limits the possibilities to learn the language and create any type of social network. Thus, as in this case, both the mother and the child become isolated, limiting their possibilities for any normal type of psychosocial development, including development of resilience.

A young man in his twenties, also among the group of “unreturnable” migrants, spoke about how the loss of rights prohibited him from living a normal life like other citizens:

*I can't marry, even if somebody liked me. We cannot marry. It's illegal. [Pause] In Norway, they know that we are here [those with rejected applications]. We have no place to go. They don't want to at least give me a work permit so I can try to go and find a job like other people. Maybe I can get a good job and work and pay tax. Even the money they give me, they can stop giving it to me [if he gets a job]*.

The participant pointed to a paradox described by many of the participants: deprivation of socioeconomic rights even though being unable to leave the country. Another paradox within this is that of forcing people to become passive recipients of money instead of allowing them to work.

### Access to Health Care

As some of the participants described above, access to a GP or access to health services in general was another concern. Health problems that were often mentioned were symptoms of anxiety, depression, difficulty to sleep, various types of physical pain, and symptoms of high blood pressure. Even though experiences varied, most of the participants described a shift from receiving necessary care, including follow-up of mental health problems, to a sudden lack of help and care after their application was refused. Among those who were “unreturnable” and with no prospect of going somewhere else in the near future, this sudden limitation of access to health care was of particular concern. A family man who was also worried about the mental health of his family members described how the refusal had affected his family:

*OBJ: We do not have access to the doctor [GP] anymore […] and because of the lack of money we cannot go to a doctor every time we have problems. If I get really ill, I will make an appointment, but then we get the bill, and it is a lot [to pay]. In addition to my mental health problems, I have a problem with high blood pressure. It seems like if I get really [mentally] ill, I also get high blood pressure*.

In this family, as in many others, the small amount of money available was prioritized for physical or emergency health problems. Thus, even though two other family members were (according to the participant) in need of mental health services, this was not being considered as a treatment option within reach, as only “emergency care” qualified for free access to health services for this group (rejected applications).

A young man in his twenties, among the group of “unreturnable” migrants, expressed how he felt “punished” or was meant to suffer, even though he was in a situation impossible to resolve:

*I am here because no one can deport me. And they can't give me papers. […] They know my country is at war, but they don't give it [residence permit] to me. They just want me to suffer until I … [pause]. Maybe they want me die in this place, because many people have died here. They got sick. I can't get medicine if am sick. Before we had a GP, but he is gone now [after the final rejection]*.

### The Double Burden of Becoming an “Adult” and Being Rejected

Several of the participants came to Norway as minors, and a striking pattern in their stories is how they experienced the transition from being defined as a minor to being defined as an adult, often in the same period as their application was rejected. Some talked about how the transition between being a minor and being an adult was an experience equally painful as receiving the rejection. An 18-year-old boy received the refusal of his application shortly after he reached 18, and he described how his life changed in a few months:

*I was first transferred to a reception center for people under 18, minors. Then I was transferred to another reception center for minors, but after one month in that place I was transferred to a place for adults in the north of Norway, because I had turned 18*.

*INT: Can you tell a little bit about the meeting with the police, UDI, the health services, or other relevant instances?*

*OBJ: Until I reached 18 it was very good. Everyone was good to me really; I was received in a very good way. […] For example, I was in need for dentist treatment which I got, and about my mental health I was in need to talk to someone professional, which I got. […] And then I had some problems with my vision, and I received glasses. Yes, I received all the help I needed*.

*INT: But only until you were 18, or?*

*OBJ: Yes. Yes, it is like a plant that gets water until it is ripe. When you are ripe, they stop to give you that water*.

*INT: How does that feel?*

*OBJ: To die is better than the life I have in Norway now [cries] To die. […] It affects you psychologically. It is like they do not see you as a human being, but some sort of slave […] Receiving the refusal is the worst thing that has ever happened to me. And the change before 18 and after18*.

In general, the young participants were particularly devastated by the lack of activities, which resulted partly from moving from a center for minors to a center for adults and partly from a rejection of the asylum application. A 21-year-old boy who came to Norway as a minor and who had received a final rejection of his application described how the whole situation was causing mental distress:

*I suffer from a lot of stress. You can understand yourself how it is to live in an asylum center, and have nothing to do, and no possibilities. And I …I have no future. So, I struggle a lot mentally due to that. At this age I should have had the possibility to attend high school and… after the education I could have become a changed person; that could contribute to the society. […] I get 900 kroner [EUR 88o] a week, and that does not allow… it is not even enough for the food I eat. […] So, it is like living in house arrest. There is nowhere to go. […] So, it is very quiet and mentally exhausting*.

The participants used metaphors like being imprisoned, becoming a slave, being in house arrest, and meant to suffer, which seem to symbolize a feeling of being punished for something they have done, and that made them deserve to be in the situation they were in.

### Losing Recognition as a Human

The long waiting process, not knowing when or if anything is going to happen, in combination with gradual socioeconomic restrictions, made many express feelings of hopelessness and lack of worth or recognition as a human being. Some expressed such feelings (as exemplified above) through suicidal thoughts. Others tried to remain hopeful and not lose their feeling of self-worth. A common way of describing the present life situation was that of being divergent from what is characteristic of being a human, including the (human) rights and opportunities one would normally hold in a society. A 20-year-old boy who came to Norway as a minor described his many struggles during the past 4 years:

*When I first came here, I went to school. But then I got my asylum application rejected twice. Then I was not allowed to go to school. They insisted that I was above 18, and they told me it was a lie that I was a minor. […] I do not live like a human; I am just alive. I have lost the taste for life. I was not seen as a human, and even though the government knows what the situation is in my country right now, they don't want to help me. I cannot go back to my country, and I do not live like a human here, so I have lost the taste… and I feel demoralized. Just being… being alive*.

He continued by comparing the treatment of humans in his home country and in Norway:

*I see no help. It is only dark and [pause]. I sit alone at home the whole day and do not attend school. I see a similarity to the dictatorship in the country I come from. A human in Norway is also mistreated, sitting the whole day only looking at the wall. […] I have nowhere to go. I have no opportunities. […] I do not see human rights here either. It is just the same, but it is a different version of what I experienced in my home country*.

This participant described how he saw the lack of human rights reflected in the treatment he received in Norway, allowing him to be in a situation with absolutely no opportunities, even though he is not in a position where he could be sent back to his home country. He expressed feelings of total powerlessness, without any influence on his destiny.

A woman in her late forties, who is similarly unable to return to her native country, revealed how she began to have mental health issues after moving to Norway. During the second interview (appeal) she felt very ill and knew that she did not manage to inspire confidence, presenting her story in an unclear and fragmented way. Like the previous participant, she described feeling like a human without worth, no one caring or making sure that her rights are protected:

*OBJ: I got rejected, but then I got this second interview. I got a chance to explain my self once more. But the time had passed, one year, then two years, and I was mentally ill. I had been hospitalized several times in this period. So, when I was interviewed the second time, they knew, they knew 100%, that I was not well, and that I was on medication. But still they did not take that into consideration, and they carried out the interview, and I did not manage to explain my situation. […] They should not be allowed to do that […] So, I got rejected the second time*.

Later in the interview she talked about how lonely she felt, as she had no family or social network in Norway. She described a perception of being invisible in the sense of no one caring or asking how she was. Regarding her mental problems, she was asked whether she had received any help or support beyond medication:

*INT: Has anyone offered you someone to talk to, like a psychologist or anyone else?*

*OBJ: No, there is no one that takes me seriously. There is no one that cares about me. I get some money to live. But no one asks how I am, what has happened or what the future is. Nothing*.

As expressed by this woman and many others, the sense of a loss of worth or recognition as a human being is connected not only to the fact that they lose the rights to participate in society through work, studies, or leisure activities, but also through the feeling of no one actually caring about their situation.

## Discussion

The findings in the overall study show that it is extremely difficult to live in an inconclusive life situation. This is the situation for most asylum seekers and refugees, and the waiting process per se has been described in detail in a previous publication (Sagbakken et al., 2020). However, as noticed among the group of asylum seekers in our material, some wait for years to receive a final answer, first living in the hope of a positive answer to the initial application and then maybe continuing with a first and second appeal. This process, as exemplified in our study, may take more than 7 years to clarify, the mean waiting time for our participants being four and a half years. This process unfolds in the context of a bureaucratic system which one does not necessarily understand, has no influence over, and where one's future destiny lies in the hands of powerful, invisible others. Furthermore, the process unfolds within time frames that are totally unpredictable and beyond the individual's control. However, for the group in question there is an added dimension: those with (finally) rejected asylum applications cannot relate hope of change to any future point in time, as they do not have the possibility to visualize whether, when or how a potential change is going to occur. Waiting for the result of an appeal gives some sort of hope, but those with a final rejection do not have such a future point of time or expected occurrence to relate to. The gradual loss of rights, opportunities and financial support occurs in the same phase as the hope of receiving a positive answer to the application of residence permit diminishes. As illustrated through the findings, being in such a situation is characterized by an increasing sense of existential emptiness, powerlessness, and hopelessness.

### Waiting for Nothing

A direct result of the hopelessness is manifested in the daily struggle to pass the time, a description mirrored in the interviews in the present study. For some of the participants, hope was constructed through personification (authorities with power) and the potential for a reversed decision, while for others through suicidal ideations or suicide as a kind of salvation. The latter must also be seen from a developmental perspective, as suicidal ideations are not uncommon in late adolescence and can end in actual suicide (Gvion and Fachler, 2017), suicide being a major cause of death in late adolescence (Pelkonen and Marttunen, 2003). Many of the participants in our study are in this age group, and living as asylum seekers is obviously an unfavorable context for adolescent development and will probably increase the risk for self-harm and suicide (Varvin et al., 2021). A Norwegian study focusing on how unaccompanied minor asylum seekers cope with temporariness found that those that had received a temporary residence permit or had been rejected considered the system and the environment as hostile. They found that they had few resources and little power to influence their situation, and there were several examples of self-destructive reactions such as self-harm and suicide attempts (Valenta and Garvik, 2019). Higher risk of self-harm and suicide among asylum seekers and refugees has been documented in several studies, although the research here is insufficient (Vijayakumar, 2016; Morrow and Krishna, 2019; Gargiulo et al., 2021). The background for self-harm and suicidal ideations is complex, but in this context it must be seen in connection with disrupted normal development, loss of contact and relationships with family and primary objects, and lack of age-adequate support (Gvion and Fachler, 2017). The pain inherent in statements such as “just being alive” signifies not only a lack of daily or future-oriented activities, but also a position in which one has nothing to expect in the future. Previous studies show that a major source of insecurity and powerlessness is the tension between anticipating time ruptures through frequent changes, such as moving to a new asylum center or being refused a residence permit (described by participants in this study as being “thrown down a mountain” or suddenly becoming “a plant not receiving water”), and long and indefinite periods of inactivity, uncertainty, and feeling unsafe (Griffiths et al., 2013). Separately or combined, “suspended time” (lack of change/waiting) and “temporal rupture” (dramatic shifts) can create both social and mental chaos (Griffiths et al., 2013). Feeling unsafe also expresses the often extreme lack of age-adequate support from close ones, and represents a background for understanding the overrepresentation of mental health problems found in studies of individuals with a refugee background compared to the majority population (Fazel et al., 2005; Bogic et al., 2015; Li et al., 2016), even after many years in the host country (Vaage et al., 2010). A prospective cohort study of asylum seekers in Sydney, Australia sought to examine the trajectory of trauma-related psychiatric symptoms and disability amongst asylum seekers over the course of the refugee determination process. Sixty-two of 73 asylum seekers were retained at follow-up. The accepted (16) and rejected (46) groups did not differ on pre-migration trauma or baseline psychiatric symptoms. After the decision, however, the accepted group showed substantial improvements in posttraumatic stress disorder, anxiety, depression and in mental health functioning, whereas the rejected group maintained high levels of symptoms on all psychiatric indices (Silove et al., 2007).

Being able to endure what can be described as “suspended time” becomes increasingly difficult due to the socioeconomic restrictions that follow the status of those who have no residence permit. We see how the ability to develop and otherwise socially interact to pass time is limited or non-existent in our study, partly due to the loss of opportunities to work, study, or even attend kindergarten, and partly due to a lack of financial resources that prevent access to social arenas, travel, or participation in organized activities. As a result of being barred from these arenas, one is precluded from learning the local language as well as the community's social codes, in other words, key aspects of citizenship. In a study of Syrian refugees in Germany, learning German and being allowed to work or study were stated as crucial ways of coping. In general, availability of a social network was stated as the most important source of support and coping since a social network facilitated feelings of belonging and hope. Conversely, lack of support and isolation were associated with poorer mental health (Renner et al., 2020).

As exemplified by some of the shared stories in this study, the situations in which the participants find themselves are also of an existential character. Participants expressed how the lack of social interaction and associated care makes one invisible as a human being, losing sources to confirm one's identity and life story. This is especially important in the identity-forming years of adolescence, particularly as refugees meet the challenges of acculturation together with the normal developmental tasks of adolescence (Streeck-Fischer, 2019; Opaas et al., 2020).

### Social Suffering Through Structural Violence

Another lens in which these findings can be viewed is through the concept of *social suffering*, developed to capture whole life narratives instead of the individualized and medicalized perspectives of psychology and psychiatry (Kleinman and Kleinman, 1996; Kleinman et al., 1997). Social suffering is seen as a product of the political, social, and cultural contexts in the sense that one acknowledges that socioeconomic and socio-political forces can at times be the direct or indirect cause of diseases and illnesses. Furthermore, the theory of social suffering eliminates the historical distinction between what is a health problem and what is a social problem by framing conditions that are both, and that require both health and social policy measures (Kleinman et al., 1997). The suffering the participants in our study describe can be explained as the result of the influence of both socioeconomic and socio-political forces, since such forces inhibit the possibility for social, physical, and mental growth and wellbeing in a group with little influence or power. Thus, we may claim that these individuals are exposed to structural violence, a concept describing a form of violence wherein some social structure or institution may harm people by preventing them from satisfying basic needs (Galtung, 1969, p. 168).

The narratives presented by participants in our study reveal that they lose not only the possibility to obtain citizenship, but also the right to work, education and social benefits. These losses lead to a series of further losses, such as access to social arenas, and thus, the ability to meet, interact and communicate with other human beings. Living in the frame of a financial situation that barely covers an existential minimum and that only covers emergency health care needs reinforces the possibilities to realize somatic, social, and mental needs. People living in a situation like this may socially and mentally remain in what can be described as limbo or a liminal phase. These concept has been used as theoretical metaphors in anthropological and sociological studies describing the process of migrant settlement, a territorial passage that marks the transition from one way of life to another through three phases: separation from the known social group or society, the transition phase (liminal phase), and incorporation into a new social group or society (Turner, 1964; Chavez et al., 1989; Chavez, 1994, 2012). In the case of our participants, they are in a situation where they have lost their connection with their culture and original status as citizens of their country without receiving a new, clarified identity as citizens of Norway. In contrast to others in similar phases (for example, prison inmates serving a defined sentence), a person who has been rejected and is unable to return to their home country will not know whether he or she will ever enter a new, clarified phase of life.

### Structural Violence as a Human Rights Violation

The Universal Declaration of Human Rights (United Nation's General Assembly, 1948) is a milestone document that secures human rights throughout the world. A central point is that all humans are entitled to citizenship, and as a member of a society the right to social security and “realization of the economic, social and cultural rights indispensable for his dignity and the free development of his personality” (Article 22). Another central point encompasses the right to work and the right to education (Articles 23 and 26). Furthermore, several of the central human rights, such as the right to education (Article 109) and the right to work (Article 110) were incorporated into the Norwegian constitution in 2014 (The Lovdata Foundation, 2014). In other words, allowing individuals to live without the possibility to realize these rights—and without the possibility to visualize a future that involves the possibility of any personal development—is not in accordance with the Norwegian constitution nor with the Universal Declaration of Human Rights. Life situations that are so difficult that one cannot manage without psychotropic or sleep medication, and where one considers “salvation” through ending one's life, are manifestations of the severity of these violations. Based on our study, as well as on others (Chavez, 2012; Jovic, 2018), it seems legitimate to argue that living under such conditions is equivalent to severe and long-lasting social suffering due to avoidable violence imposed by external others (Galtung, 1969).

### The Regulation of Bodies

What are the mechanisms or rationale behind the practices that institutionalize social suffering and that create substantial inequalities between members of a society? Or, in other words, systematically denying members of a society *citizenship*, defined by Marshall (1950) as being a “full member of society”. One of the explanations why such suffering seems to be widely accepted may be that the structural violence to which this group of people are exposed is silent and does not necessarily show. While personal, direct, physical violence is manifest and visible, and provokes reactions from other members of a society, structural violence is indirect and may be something *natural* (Galtung, 1969). As expressed by Galtung (1969, p. 173): “Structural violence is silent, it does not show—it is essentially static, it *is* the tranquil waters”. In line with Galtung, focusing on the importance of social justice, hidden agency, and the invisibility of certain forms of violence, Nixon (2011) introduced the concept of “slow violence”. Instead of focusing on static connotations, he foregrounds questions related to temporality, to the effect not only of violence caused by invisible structures, but violence enacted slowly over time. Time becomes an actor because of the unequal attention paid to what Nixon (2011) describes as “spectacular and unspectacular time” (p. 6). In an age where people draw attention to and acknowledge “instant spectacle” (Nixon, 2011, p. 6), slow violence becomes unnoticeable due to the lack of temporal, instant, and thus special, effects. Subsequently, slow violence may easily increase due to subtle changes over time—such as a gradual loss of human rights—making it imperceptible. Furthermore, the working of time makes it easier to decouple the violence from its original cause or (lack of) justification (Nixon, 2011, p. 10–11).

How such suffering becomes a *natural* dimension in a society may—besides being (partly) invisible—be explained by the concept of biopower, a term coined by Michel Foucault to illustrate the way in which political governance increasingly seeks control of bodies and populations. Foucault (Foucault, 1978; Foucault and Rabinow, 1997, p. 73–80) draws attention to what he sees as a historical shift among liberal nation states in their increasingly use of power to protect and manage the life of the “legitimate” population (Lemke et al., 2011). Such governmentality is part of a nation-building apparatus based on norms that seek to rationalize and legitimize inclusion and exclusion mechanisms, to identify and select those that are desirable political bodies and those that are not. Power operationalized in this way is evident in both the strategies and rhetoric of immigration control, and may be subtle in its tactics while at the same time concealing its brutal consequences (Davies et al., 2017).

Concrete actions, such as deporting people and limiting the possibilities of obtaining a residence permit (permanently or temporarily) are actions that are visible signs of the nation states' power. However, as pointed out by Mbembe (2003), political *in*actions, such as withholding essential means of life, care and access to public health, work and education, and opportunities to improve one's miserable conditions, are also means of control and power. Such inactive exercise of power may assign certain individuals the status of “the living dead” (Mbembe, 2003, p. 40), destined to suffer over a long period, or even permanently—as seen in our study. Thus, a continuous wounding of undesirable individuals, rather than an active and direct killing, can become a political tool to control certain groups of bodies by merely keeping them alive “but in a state of injury” (Mbembe, 2003, p. 21), a condition that can be seen as an extension of the concept of slow violence. The social suffering and the dehumanization which these groups of refugees endure may easily become part of *normality* and be socially sanctioned, since the actual exercise of power is concealed behind slow, gradual, imperceptible actions (Nixon, 2011) or a veil of (active) inaction (Davies et al., 2017). Subsequently, bureaucratic and time-consuming processes determining refugee status can be interpreted as an intentional production of “protracted uncertainty” (Biehl, 2015, p. 70) characterized by indefinite waiting, limited knowledge about the application process and the decision makers, unpredictable legal status, and unpredictable settlement practices (Biehl, 2015; Sagbakken et al., 2020). This prolonged uncertainty serves to demobilize, contain and “criminalize” (cast suspicion on) asylum seekers, which in turn serves to *normalize* the necessity of strict bureaucracy in order to maintain security (Biehl, 2015; Sagbakken et al., 2020). The structural violence that is exercised remains invisible and unquestioned as it becomes part of the wider and processual structures in society (Davies et al., 2017). Furthermore, this exercise of power may ultimately serve as a deliberate means to constrain onward migration, as well as potentially force some of the migrants back to their home countries as a “politics of discomfort” (Darling, 2011, p. 264) or a *body politics* (Scheper-Hughes and Lock, 1987) which in both temporal and spatial dimensions deliberately rules out any sense of security or belonging, and which determines the level of uncertainty of being an asylum seeker (Biehl, 2015; El-Shaarawi, 2015; Horst and Grabska, 2015). Thus, another result of the structural violence our participants are exposed to is constant liminality; they remain “liminals” (Chavez, 1994) without the possibility to become citizens in the new society. This may again lead to the loss of a fundamental resource—hope for the future—and thus the possibility of “future-figuration”, found to be of particular importance among individuals who are displaced (Artero and Fontanari, 2021).

Another dimension of this is that intangible factors such as bureaucratic obstacles, restrictive immigration policies, racialization and/or racism, and a general suspicion toward and fear of immigrants interact in a way that triggers psychological distress in the individual and serves to legitimize the idea of being undeserving of social services (Larchanché, 2012). Additionally, as the results of this study show, adolescents who have reached the age of 18 seem to be included in the category of those who are “less deserving”, influencing the mental health of these young people. A body politics that continues to wound individuals may serve to reproduce and reinforce the notion of illegitimacy among those who represent the “legitimate population” (Lemke et al., 2011). Thus, it also serves to strengthen the notion of not belonging and of undeserving of the same rights as other human beings, in relation both to asylum seekers who have arrived and to those yet to come (Larchanché, 2012; Bendixsen, 2020). This may extend to a discussion of whether undocumented migrants/rejected asylum seekers are morally deserving of resources such as education and health care (Larchanché, 2012); unveiling the idea of a *moral right* to distinguish between lives that are to be preserved and enhanced and lives that are not worth preserving (Bendixsen, 2020).

## Concluding Remarks

In this study, asylum seekers who are waiting for appeals or who have received a final rejection expressed their experiences of different forms of violence and suffering. This violence seems legitimized by the idea that these people do not constitute true citizens, are not part of the legitimate population, and are neither desirable nor deserving political bodies. This idea, and the subsequent practice, also applies to cases where people cannot return to their country of origin when they have no spatial place to go within any imaginable temporal frame.

This type of violence can be interpreted as a form of governance connected with nation-building, a strategy of limiting the number of undesirable political bodies. In subtle ways, it seeks to rationalize and legitimize deprivation of political rights and possibilities to realize physical, social, and mental needs—even when this policy conflicts with the Universal Declaration of Human Rights and its incorporation into the Norwegian constitution. The invisible, gradual, and slow nature of the violence facilitates detachment from the original justification and blurs the absurdity in the protracted and often unresolvable situations in which asylum seekers find themselves. Thus, violation of human rights may easily become part of *normality* since the actual exercise of power is hidden behind imperceptible actions or inactions, thereby concealing its possibly intended brutal consequences.

## Data Availability Statement

The raw data supporting the conclusions of this article will be made available by the authors, without undue reservation.

## Ethics Statement

The studies involving human participants were reviewed and approved by the Regional Committees for Medical and Health Research Ethics (REC). REC SouthEast-Secretariat: 2016/651. The patients/participants provided their written informed consent to participate in this study. Written informed consent was obtained from the individual(s) for the publication of any potentially identifiable images or data included in this article.

## Author Contributions

MS and SV were responsible for the conception of the study and development of the study design. MS was responsible for drafting the manuscript, while all authors have contributed by writing specific parts and by critically revising it. All authors participated in the data collection, data analysis, read and approved the final manuscript and are accountable for all aspects of the work in ensuring that questions related to the accuracy or integrity of any part of the work are appropriately investigated and resolved.

## Conflict of Interest

The authors declare that the research was conducted in the absence of any commercial or financial relationships that could be construed as a potential conflict of interest.

## Publisher's Note

All claims expressed in this article are solely those of the authors and do not necessarily represent those of their affiliated organizations, or those of the publisher, the editors and the reviewers. Any product that may be evaluated in this article, or claim that may be made by its manufacturer, is not guaranteed or endorsed by the publisher.
